# Prevalence and attitude towards hepatitis B vaccination among healthcare workers in a tertiary hospital in Ghana

**DOI:** 10.11604/pamj.2020.36.244.24085

**Published:** 2020-08-05

**Authors:** Elizabeth Tabitha Botchway, Elizabeth Agyare, Letsa Seyram, Kwadwo Koduah Owusu, Mohamed Mutocheluh, Dorcas Obiri-Yeboah

**Affiliations:** 1 Department of Internal Medicine, Cape Coast Teaching Hospital, Cape Coast, Ghana,; 2 Clinical Microbiology/Public Health Unit, Cape Coast Teaching Hospital, Cape Coast, Ghana,; 3 School of Medical Sciences, College of Health and Allied Sciences, University of Cape Coast, Cape Coast Ghana,; 4 Clinical Care, National AIDS/STIs Control Programme, Accra, Ghana,; 5 Department of Microbiology, School of Medical Sciences, Kwame Nkrumah University of Science and Technology, Kumasi, Ghana,; 6 Department of Microbiology and Immunology, School of Medical Sciences, College of Health and Allied Sciences, University of Cape Coast, Cape Coast, Ghana

**Keywords:** Vaccination, hepatitis B virus, healthcare workers

## Abstract

**Introduction:**

adequate knowledge on hepatitis B virus (HBV) infection is important among healthcare workers (HCWs) as this impacts the vaccination seeking behaviour. This study sought to assess the knowledge, vaccination status and related factors amongst HCWs in a tertiary facility in Ghana.

**Methods:**

an analytical cross-sectional study was conducted amongst full-time HCWs of different categories at the Cape Coast Teaching Hospital, Ghana. Stratified sampling was used to arrive at the number needed for each category of HCW and then simple random sampling to recruit participants. A structured self-administered questionnaire was used. Descriptive statistics and logistics regression were carried out on the data.

**Results:**

a total of 303 HCWs participated with 78.07% (n=235) being between 20-30 years, and majority being females (62.38%, n=189). A total of 186 (61.39%) participants had adequate knowledge, mean knowledge score was 4.73/7 (±0.97). About 80% (n=218) had received the 3 doses of HBV vaccine. Among the unvaccinated, cost was the major barrier (62.07%, n=18). Participants who did not know that HBV was more infectious than HIV (aOR=5.31, 95%CI: 1.91-14.77), p<0.001) and those who did not have knowledge that HBV vaccine was effective were more likely to be unvaccinated (aOR=8.63, 95%CI: 2.99–24.94), p<0.0001). The gender and cadre of staff did not show statistical evidence of an association with vaccination status.

**Conclusion:**

knowledge on HBV is paramount for all HCWs as well as the importance of receiving the full doses of the hepatitis B vaccines. Barriers to vaccination must be removed to ensure protection of HCWs.

## Introduction

Hepatitis B virus (HBV) infection remains one of the most important infections affecting people in sub-Saharan Africa. The World Health Organisation (WHO) estimated in 2015 that about 257 million people were living with chronic hepatitis B virus most of whom were living in the African and Western Pacific Regions [1]. HBV is a major cause of liver cirrhosis and hepatocellular carcinoma (HCC) which is a major cause of mortality in the world and in sub-Saharan Africa [2]. The prevalence of HBV infection is estimated to be greater than 8% and thus makes Ghana a country with a high prevalence. A meta-analysis done in 2016 placed the estimate of HBV infection at 12.3% in Ghana [3]. Hepatitis B virus infection is however, a vaccine preventable illness and the WHO has made accessible the 3-dose vaccine that helps in reducing infections and subsequently reduce mortality associated with the disease [1, 2]. Some systematic studies have demonstrated the efficacy of the universal vaccine coverage, with adolescents demonstrating 76% lower prevalence of hepatitis B virus infection [4]. In South Africa, the prevalence of occult HBV infection reduced from 70.4% to 66% with the introduction of universal vaccination [5]. High-risk groups like healthcare workers (HCWs) and children born to HBV positive mothers are important target groups in the vaccination programme [2].

The knowledge of HCWs on the virus, its infectivity and the vaccination strategy are particularly important as they are the group that will help impart knowledge and aid in the improvement of vaccination strategy in the general population. The level of knowledge in HBV infection among HCWs have been demonstrated in certain jurisdictions to be generally low in Africa; for example, 38.8% in Freetown, Sierra Leone, 56.7% in Lagos state in Nigeria and 62% in North-West Ethiopia [6-8]. Studies have shown that in some parts of Ghana and other countries, as much as 50% of health workers were not vaccinated against HBV [9, 10]. The reasons for non-vaccination included inability to afford the vaccine, distrust of vaccination in general and some concerns about vaccine safety [6, 9]. It is essential for countries and health facilities to determine the situation of the HCWs as far as vaccination against such infectious pathogens are concerned. This will enable targeted inventions at the local level and add to the data at the national level to impact policy which will ensure protection of these at-risk population of HCWs. This study therefore assessed the knowledge, vaccination status and the related factors amongst HCWs at the Cape Coast Teaching Hospital (CCTH) to identify any barriers and/or gaps to tailor interventions appropriately.

## Methods

**Study design and population:** the study was an analytical hospital-based cross-sectional study conducted in the second quarter of 2019 among HCWs at the CCTH, a tertiary healthcare institution in the central region of Ghana. It was carried out among doctors, nurses and other healthcare workers who worked full time at the facility. Written informed consent to participate in the study were first obtained. Students and health professionals who were on internship were excluded from the study. CCTH had a total population of 912 full-time healthcare workers consisting of 209 doctors, 661 nurses and 42 other paramedical staff (e.g. pharmacists, biomedical scientists, physiotherapists etc.) at the time of this study. Using this total population, the calculated sample size required was 278 participants which was further stratified to 63 doctors, 201 nurses and 14 other HCWs. Participants were recruited using simple random sampling (picking the yes or no) and were given a questionnaire to fill which were then collected. Making room for non-response and missing data, a total of 332 questionnaires were distributed. The questionnaire was developed based on the specific objectives set for the study and was pretested in a group of twenty (20) participants from the target population to help refine and clarify it before being used for the actual study. These participants were excluded from the main study. The questionnaire sought to ascertain some socio-demographic characteristics of the participants. It then sought to determine basic knowledge concerning HBV, HBV vaccination status and associated factors among these HCWs.

**Ethical considerations:** approval for the study was sought from the CCTH ethical review committee (Ref: CCTHERC/EC/2019/01>6). The study objectives were explained to the respondents and informed consent was sought from them. The questionnaires were anonymised, and respondents were assured they can withdraw from the study at any time.

**Data processing and analysis:** the data collected were checked, coded, and captured using Microsoft Excel spreadsheet and then it was cleaned and analysed using STATA version 14 (STATA Corp, Texas USA). Knowledge items were given scores and participants scores were summed up. The highest score expected was 7 and minimum of 0. Any score ≥5 was considered as adequate knowledge score and any score <5 was inadequate. The data analysis began with descriptive statistic using frequencies and percentages calculated with appropriate measures of central tendencies and were presented in tabular summaries. This was followed by bivariate and multivariate analysis and presented with p-value of 0.05 interpreted as statistical evidence of association between variables. Variables with p-values <0.2 from the univariate analysis were included in the model for multivariate analysis.

## Results

**Socio-demographic characteristics of study participants:** more females (62.38%, n=189) were enrolled into the study and 78.07% of participants were between 20-30 years (n=235). In the study, nurses/midwives comprised a higher percentage of the cadre of staff at 66.3% (n=201), followed by doctors at 23.4% (n=72) and the others 9.9% (n=30) which is in line with the staff mix of the hospital (Table 1).

**Table 1:** socio-demographic and other relevant characteristics of study participants (N=303)

Variable	Mean/Frequency (n/n)	SD/Percentage (±%)
**Age, years (N=301)**		
Mean	28.70	4.54
20-30	235	78.07
31-40	56	18.6
>40	10	3.33
**Sex**		
Male	114	37.62
Female	189	62.38
**Occupation**		
Doctors	72	23.76
Nurse/Midwife	201	66.34
^*^Others	30	9.9

* These comprised pharmacists, laboratory technicians/technologists and health assistants

**Knowledge on HBV among healthcare workers:** all the participants had heard about hepatitis B virus (HBV) infection. A total of 186 (61.39%) participants had adequate knowledge and the mean knowledge score was 4.73 (±0.97). However, 48.51% (n=147) thought that the infectiousness of HBV was either same as the human immunodeficiency virus (HIV) or less infectious than HIV. Most participants knew the medium through which HBV is transmitted; with 86.8% (n=263) asserting that HBV was transmitted through blood, 87.3% (n=264) through body fluids contaminated with infected blood, 65.02% (n=197) for both saliva and sweats. None of the participants thought HBV risk to HCW could be classified as low risk. In contrast, n=271 (89.44%) participants thought the risk could be classified as high risk (Table 2).

**Table 2:** basic knowledge on hepatitis B virus among study participants (N=303)

Variables	Mean/Frequency (n)	Percentage (%)
**Heard about hepatitis B virus infection?**		
Yes	303	100
No	0	0
**How infectious do you think hepatitis B virus is compared to HIV?**		
Less than HIV	16	5.28
Same as HIV	131	43.23
More than HIV	151	49.87
I don´t know	5	1.65
^*^HBV is effectively transmitted through contact with…?		
Blood	263	86.8
Saliva	197	65.02
Sweat	197	65.02
Body fluids contaminated with infected blood	264	87.13
**Level of risk of getting HBV infection as Health care workers**		
No risk	2	0.66
Low risk	0	0
Moderate risk	25	8.25
High risk	271	89.44
I don´t know	5	1.65

* Multiple responses allowed

**Knowledge and attitude towards HBV vaccination among HCWs:** all the participants had heard about HBV vaccination. 88.5% (n=268) responded that the HBV vaccine was very effective. However, n=7 (2.3%) of the participants did not know how effective the vaccine was. An impressive 95.4% (n=289) asserted that the recommended full dose of the vaccine was 3 doses, although 4.3% (n=13) still did not know what the recommended full dose was. There was diverse knowledge on the length of protection after HBV vaccination with the highest being 5 to 10 years (48.5%, n=147). Also, 90.4% (n=274) of the participants had received HBV vaccination out of whom almost 80% (n=218) had received 3 doses of the vaccines. After vaccination, only 40.9% (n=112) had tested to verify sero-protection status (Table 3).

**Table 3:** knowledge and attitude towards HBV vaccination among study participants

Variables	Frequency (n)	Percentage (%)
**Have you ever heard about HBV vaccination?**		
Yes	303	100
No	0	0
**Is HBV vaccine effective?**		
Not effective	0	0
Slightly effective	28	9.24
Very effective	268	88.45
I don´t know	7	2.31
**What is the recommended full dose of HBV vaccine?**		
1	0	0.00
2	1	0.33
3	289	95.38
I don´t know	13	4.29
**Duration of protection from full HBV vaccination (years)**		
<5	85	28.05
5 to 10	147	48.51
>10	44	14.52
I don´t know	27	8.91
**Have you received HBV vaccination?**		
Yes	274	90.43
No	29	9.57
**Doses of HBV vaccine have received (N=274)**		
1	3	1.09
2	33	12.04
3	218	79.56
>3	20	7.3
**How long since receiving last dose of HBV vaccination (months)**		
<3	16	5.84
3 to 6	35	12.77
>6	223	81.39
**Have you tested to prove sero-protection since your vaccination?**		
Yes	112	40.88
No	162	59.12

**Figure 1:**
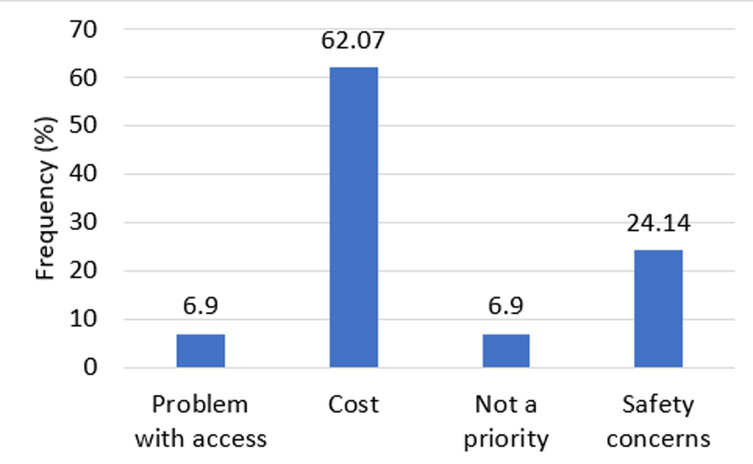
reasons for not being vaccinated against HBV (N=29)

**Factors associated with HBV vaccination status:** cost was a major factor that hindered people from being vaccinated against HBV infection (62.07%, n=18). Participants also cited safety concerns as a reason for being vaccinated (24.14%, n=8) whiles problems with access (6.9%, n=2) and the vaccination not being a priority were part of the reasons cited (Figure 1). Multivariable analysis reveals that age of participants was associated with vaccination status with those older than the mean age of 28.8 being less likely to be unvaccinated (aOR=0.11, 95%CI: 0.04-0.28), p<0.0001). Those who did not know that HBV was more infectious than HIV were more likely to be unvaccinated (aOR=5.31, 95%CI: 1.91-14.77), p<0.001). In addition, those who did not have the knowledge that the HBV vaccine has proven effectiveness were more likely to be unvaccinated (aOR=8.63, 95%CI: 2.99-24.94), p<0.0001). The gender, cadre of staff and knowledge on the level of risk posed by HBV to HCWs did not show statistical evidence of an association (Table 4).

**Table 4:** univariate and multivariate analysis of vaccination status among participants (N=303)

Variable	OR (95% CI)	P-value	aOR (95% CI)	P-value
**Mean Age (years) N=301**				
<mean age	1		1	
>mean age	0.16 (0.07-0.38)	<0.0001	0.11 (0.04-0.28)	<0.0001
**Gender**				
Male	1			
Female	1.62 (0.75-3.51)	0.213		
**Cadre of staff**				
Doctor	1			
Nurse/Midwife	0.58 (0.21-1.59)	0.281		
Others	2.16 (0.24-19.67)	0.482		
**How infectious do you think HBV is compared to HIV?**				
More than HIV	1		1	
All other responses	2.87 (1.21-6.76)	0.012	5.31 (1.91-14.77)	0.001
**Level of risk of getting HBV infection as health care workers**				
High risk	1			
All other responses	2.49 (0.92-6.71)	0.062		
**Is HBV vaccine effective?**				
Very effective	1		1	
All other responses	4.29 (1.74-10.60)	<0.0001	8.63 (2.99-24.94)	<0.0001

Adjusted for age, knowledge on the level of infectiousness of HBV compared with HIV and knowledge on effectiveness of the HBV vaccine in multivariable analysis

## Discussion

The World Health Organization (WHO) aims at eliminating viral hepatitis as a major public health concern by 2030 and knowledge of the virus among the populace is a major step in eliminating this threat [1]. Healthcare workers (HCWs) form part of the major stakeholders in this drive. In this study, we sought to determine the level of knowledge about HBV and vaccination rate among HCWs in a tertiary facility in Cape Coast, Ghana and factors associated with vaccination. In general, HCWs in this study which comprised doctors, nurses and other cadres of staff, had adequate basic knowledge about HBV infection. However, there were still gaps in the knowledge about the infectiousness of HBV as a total of 48.5% presumed that it was either less or equal to HIV in level of infectiousness. Despite several factors such as the genetic make-up, viral load, mutation, viral infection stage and binding of virus to endogenous neutralizing antibodies influencing the infectivity of HBV and HIV, Kleinman *et al*.in 2009 demonstrated through modelling that HBV transmission through blood transfusion is higher than that of HIV infection, with the possibility of people with occult HBV infection transmitting the virus [11]. The knowledge on the route of transmission of HBV was high (87.13%) for body fluids contaminated with blood. Targeted education of HCWs possibly as part of in-service training is still required to increase this knowledge base among HCWs to ensure that the knowledge about routes of transmission is adequate [12]. A study carried out in Ghana among nurses found knowledge on pre- and post-exposure strategies to prevent HBV infection to be poor [13]. Other studies have also found poor knowledge scores among HCWs in Sierra Leone, Ethiopia and among students in health professional training institutions [10, 14]. In a study conducted by Ochu *et al*. in Nigeria, 42.2% (144/341) of HCWs had adequate knowledge on HBV [15]. Increasing knowledge and awareness among HCWs have thus been proposed to improve vaccination coverage in similar settings in Ghana [13, 16]. In this study knowledge about the infectiousness of HBV, the effectiveness of the vaccination was positively correlated with respondents being vaccinated against HBV.

The WHO stipulates that every person who is non-immune should receive 3 doses of hepatitis B vaccine at 0, 1 and 6 months [1, 17]. Sero-protection is recommended to be tested 1-2 months after vaccination especially for such high-risk populations like HCWs. In this study majority of the HCWs knew the recommended full dose vaccination schedule for HBV infection and had received it accordingly (90.4%, 86.9% respectively). This rate of vaccination is higher than found among HCWs in a study in Accra. A study in Geuteng Province in South Africa reported that only 19.9% of HCWs were fully vaccinated [18]. In China, it was found that 60% of HCWs had received > 3 doses of the HBV vaccine [19]. However, more than half of the respondents (59.12%) had not tested for sero-protection post vaccination. Obiri-Yeboah *et al*. in another study among 711 HCWs found that among the vaccinated HCWs, 1% (n=7) were infected and 8.2% (n=58) did not have adequate sero-protection [20]. This is an important area of knowledge sharing as HCWs must be educated after getting vaccinated, it will be useful if the person tested to ensure he/she has adequate antibody titres to fight against the virus. Among the 29 (9.6%) who had not been vaccinated in this study; cost, safety concerns, and access were the reasons given. In Ghana, and most countries in sub-Saharan Africa, HCWs are expected to still pay for the HBV vaccine if they are to receive it. In addition, there is need to address the safety concerns as expressed by some.

A metanalysis by Auta *et al*. demonstrated that cost, unavailability of the vaccine and busy work schedule were some of the reasons for HCWs not seeking vaccination against HBV. This was similar in South-West Cameroon, where cost of vaccination, fear of injection and ignorance were mentioned [21, 22]. It is therefore essential that these barriers be removed or minimized for all HCWs and the general population to be able to get vaccination as needed. The continuation of HBV vaccination as part of the childhood vaccinations and efforts to improve the uptake will also contribute to a generation of people including future HCWs who are vaccinated and protected. This study was carried out in only 1 tertiary health facility and hence might not represent the situation among HCWs in the country. It does not also cover all possible areas of knowledge assessment on HBV infection. Despite these limitations, it gives a window into the situation and adds to the accumulating evidence on the level of vaccination among HCWs and its related factors.

## Conclusion

This study highlights the importance of improving the knowledge of HCW on HBV. In addition, other barriers to vaccination such as cost must be removed for such high-risk populations.

### What is known about this topic

- Knowledge of HCW impart the dissemination of vaccination processes of the rest of the population;
- The level of knowledge on hepatitis B virus infection amongst HCWs in Africa is generally low.

### What this study adds

- The vaccination rate among healthcare workers is still low in even tertiary level facility as this study site;
- Among the vaccinated, there is inadequate knowledge on the need to determine and confirm post vaccination sero-protection through antibody titre test;
- Barriers including cost and safety concerns remains as a barrier for uptake of vaccination by HCWs.
